# Placental miRNA expression in association with in utero particulate air pollution exposure

**DOI:** 10.1186/2049-3258-73-S1-P36

**Published:** 2015-09-17

**Authors:** Maria Tsamou, Karen Vrijens, Narjes Madhloum, Wouter Lefebvre, Charlotte Vanpoucke, Wilfried Gyselaers, Tim S Nawrot

**Affiliations:** 1 Hasselt University, Diepenbeek, Belgium; 2 Flemish Institute for Technological Research (VITO), Mol, Belgium; 3 Belgian Interregional Environment Agency (IRCELINE), Brussels, Belgium; 4 Department of Obstetrics, East-Limburg Hospital, Genk, Belgium

## Background and aims

Particulate matter exposure during *in utero* life may entail adverse health outcomes later in life. Epidemiological studies in adults have linked air pollution's adverse effects to alterations in gene expression profiles, which can be regulated by epigenetic mechanisms, including microRNAs (miRNAs). MiRNAs have been implicated in diverse biological processes. We investigate the potential influence of air pollution exposure in early life on placental miRNA expression.

## Methods

Within the framework of the **ENVIR*ON*AGE** birth cohort, the expression of four miRNAs (miR-16, miR-21, miR-146a and miR-222) was analyzed by qRT-PCR in placental tissue from 211 mother-newborn pairs. Multiple regression models were used to study placental miRNA expression and *in utero* exposure to particulate matter over various time windows during pregnancy. *In silico* analysis was performed to predict genes and pathways targeted by the studied miRNAs.

## Results

All four measured placental miRNAs were associated with air pollution exposure in early-life. For each 5 μ/m^3^ increase in PM_2.5_ exposure, the expression of miR-21, miR-146a and miR-222 was reduced by 32.1% (95%CI: -52, 3.8, p=0.0305), 30.1% (CI: -47.3, -7.1, p=0.0144) and 23.9% (CI: -41.8, -0.6, p=0.0462) during the 2^nd^ trimester, respectively. The effects were independent of mother's age, pre-gestational BMI, smoking status, parity and educational status, and newborn's gender and gestational age, seasonality and apparent temperature. Pathway analysis based on in silico predicted miRNA targets revealed immune responses as the core pathways targeted by the studied miRNAs.

## Conclusion

Environmental exposure to particulate air pollution in early-life can modify the placental expression of miRNAs-21, -146a and -222 in human placental tissue. These miRNAs might be relevant targets for PM induced effects in fetal programming and could potentially lead to health outcomes later in life.

